# How holobionts get sick—toward a unifying scheme of disease

**DOI:** 10.1186/s40168-017-0281-7

**Published:** 2017-06-24

**Authors:** Silvio D. Pitlik, Omry Koren

**Affiliations:** 1 Department of Molecular Genetics, Weizmann Institute of Science, Rehovot, Israel; 2 Faculty of Medicine, Bar Ilan University, Safed, Israel

**Keywords:** Microbiome, Disease, Pathobiome, Pathobiont, Microbiota

## Abstract

All humans, animals, and plants are holobionts. Holobionts comprise the host and a myriad of interacting microorganisms—the microbiota. The hologenome encompasses the genome of the host plus the composite of all microbial genomes (the microbiome). In health, there is a fine-tuned and resilient equilibrium within the members of the microbiota and between them and the host. This relative stability is maintained by a high level of microbial diversity, a delicate bio-geographic distribution of microorganisms, and a sophisticated and intricate molecular crosstalk among the multiple components of the holobiont. Pathobionts are temporarily benign microbes with the potential, under modified ecosystem conditions, to become key players in disease. Pathobionts may be endogenous, living for prolonged periods of time inside or on the host, or exogenous, invading the host during opportunistic situations. In both cases, the end result is the transformation of the beneficial microbiome into a health-perturbing pathobiome. We hypothesize that probably all diseases of holobionts, acute or chronic, infectious or non-infectious, and regional or systemic, are characterized by a perturbation of the healthy microbiome into a diseased pathobiome.

## One sentence summary

We hypothesize that probably every illness of holobionts is characterized by some perturbation of the microbiome/microbiota into a pathobiome.

## Main text

For more than a century, diseases of humans have been classified in a dichotomous fashion as infectious and non-infectious. In the former group, causality has been framed in the context of Koch’s postulates, in most cases linking a single microbe with a single disease. This approach, although incomplete from a patho-physiological point of view, has led to enormous advances in the prevention and treatment of infectious diseases. During the last decade, a huge amount of data has been gathered on the composition of the human microbiota in health and disease. Nowadays, almost no sickness has been overlooked by this trend, and there is expanding information on the complex configuration of the human microbiota in almost every illness. Interestingly, this knowledge includes not only non-infectious chronic diseases such as obesity, diabetes mellitus, inflammatory bowel disease, and many others but also a growing list of infectious diseases that were traditionally ascribed to a single pathogen behaving as a lone warrior against the host. For example, in AIDS, a disease caused by the HIV virus, a perturbed microbiota is found in the vagina of women with a higher predisposition to contract the infection [1], in the gut of patients already infected with HIV [2], and also in HIV-negative neonates born to mothers who received successful anti-retroviral therapy [3]. As an additional example, *Staphylococcus aureus* lives as a dormant pathobiont in the nares of more than a third of healthy individuals. Recent investigations have shown that this bacterium remains confined to the nares due to an intricate interaction with other organisms. For example, the nearby presence of *Staphylococcus lugdunensis* kills *S. aureus* through a newly discovered antibiotic termed lugdunin [4]. On the other hand, the adjacent growth of *Corynebacterium striatum* and *S. aureus* results in diminished expression of virulence genes of *S. aureus* but augmented expression of genes involved in non-virulent colonization [5]. Switching roles from defenders to attackers, *S. lugdunensis* or *C. striatum* may cause serious infections [6, 7]. *S. aureus* cohabitating other body sites, for example, the lungs, may increase the virulence of other microbes in the vicinity [8]. *Escherichia coli*, a major member of the gut microbiota, may cause life-threatening infections. Conversely, probiotic strains of the same bacterium may prevent recurrence of bladder infections in women [9] or the development of rampant cholera infection in experimental settings [10]. Many other microbes, acting as endogenous or exogenous pathobionts, may appear to act as independent aggressors, but their ability to trigger disease is actually orchestrated and regulated by members of the microbiome/microbiota. For example, carriage of the arbovirus is influenced by the microbiota of its mosquito vector [11] and attraction of biting mosquitoes is dependent on the individual’s skin microbiota [12].

**Fig. 1:**
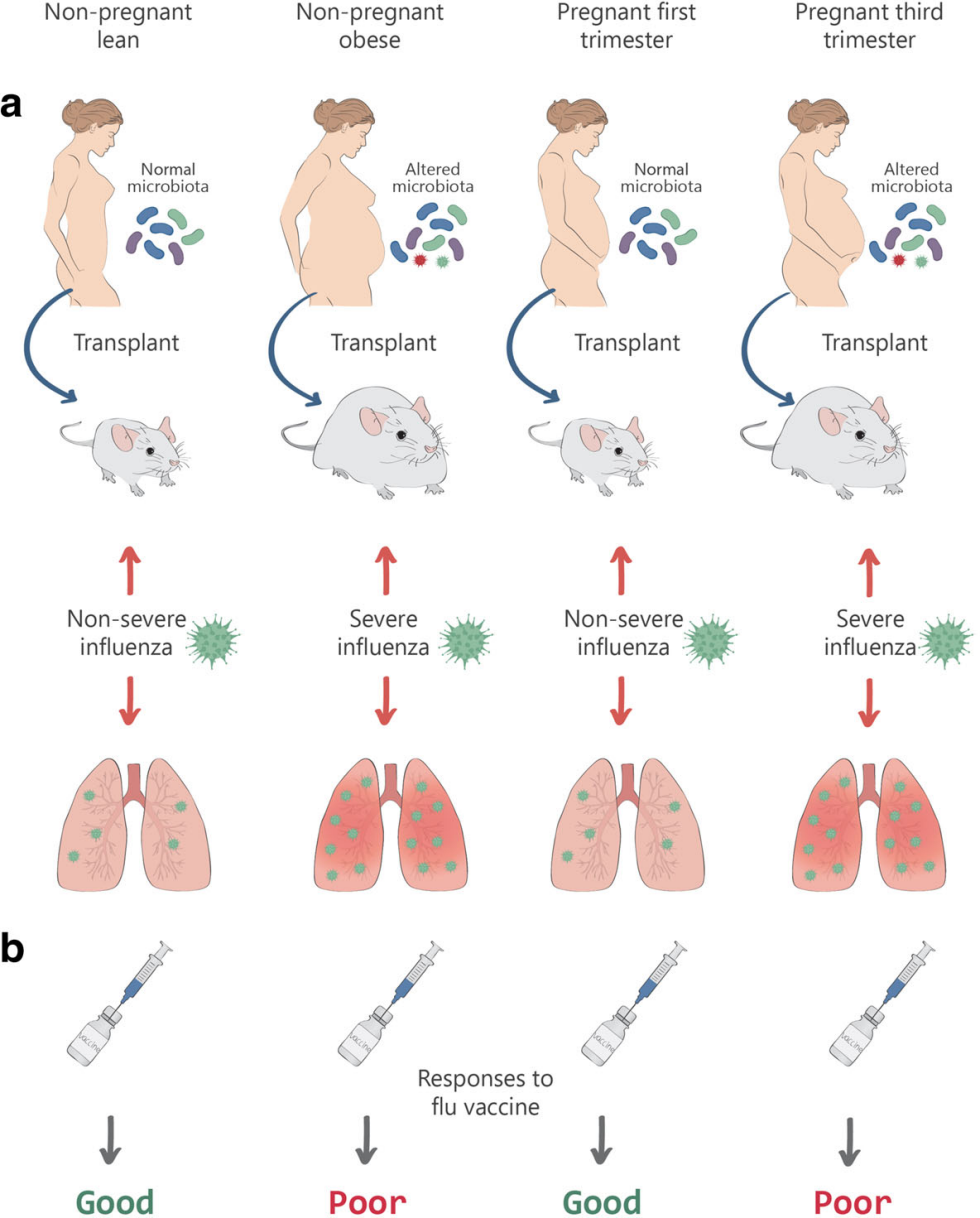
What is a good microbiome? A typical example of the tripartite interaction between a common virus, the host, and the microbiota. **a** Some strains of the influenza virus cause more severe disease in obese persons or mice as well as in third-trimester pregnant woman. This occurrence correlates with a disturbed gut microbiota. **b** In addition, suboptimal responses to the flu vaccine are found in these populations

We are still in a difficult position to optimally define a “normal” or “healthy” microbiota. As in ecological niches, there are multiple states of equilibrium corresponding to healthy states. For example, some of the biomarkers for dysbiosis include an increase in beta (between sample) diversity, a decrease in alpha (within sample) diversity, an increase in the abundance of members of Proteobacteria and opportunistic pathobionts, and an increase in inflammatory markers. As summarized in Fig. 1a, these changes happen not only in obese individuals (“bad” or “diseased” microbiome) but also in women in their third trimester (“good” or “healthy” microbiome) [13, 14].

Interestingly, an adverse outcome of influenza and a suboptimal immunogenicity of the flu vaccine are influenced by these conditions, in humans and experimental animals (Fig. 1) [15–18]. Similar interactions between pathobionts, the microbiota, and the host are increasingly being described in animals and plants [19–21]. In sick corals, there is a significant change in the ranking order of viruses that are also present in healthy specimens but with a distinct load for each type of virus [22]. In light of this paradigm, we suggest that novel ways to establish complex causation, as opposed to associations between a perturbed microbiota and a sick host, should be developed. Under this scheme, elucidation of causality becomes a very difficult task which can be compared metaphorically to untying a Gordian knot. Alternatively, we can apply an ecological approach and recognize that disease affects not the host but the holobiont in all its complexity. When doing research on the microbiota, we are actually dealing with an intricate system composed by microbial individuals interacting in a social fashion. Consequently, when trying to understand holobionts in health and disease, we may apply novel methods developed by social and economic scientists [23]. Relatively simplified biomarkers for selected diseases may be looked for and calculated by complex computational methods based on parameters such as microbial diversity of the microbiota, predominance or absence of specific species of bacteria, levels of defined metabolites, and other.

Words have an extraordinary power [24]. Some (like *pathogen* or *pathogenicity*) may obscure the right interpretation of biological processes, and other words may facilitate the clarification of complex developments [25]. We claim that the introduction of certain terms to the world of Medicine may greatly improve our understanding of both health and disease. These words include *holobionts*, *pathobionts*, and *pathobiome* (a disturbed or dysbiotic microbiome).

The current classification of diseases into infectious and non-infectious should be revised. While there may be one or more pathobionts playing a central role in the former case, there is a concurrent perturbation of the microbiota which can either precede or follow the introduction of the pathobiont/s into the holobiont system. The diagnosis of classic infectious diseases is performed by well-established methods including microscopic observation of a specific microbe, culture-dependent and culture-independent molecular techniques such as PCR, or the detection of specific antibodies as a result of an active infection. Still, under this approach, important questions remain unanswered. For example, one might ask why certain hosts develop disease, while others remain healthy. In contrast, dysbiotic or perturbed states of the microbiota are not routinely evaluated in clinical practice due to their complexity and the scarcity of simple ways to characterize them. The multiplicity of co-factors and the slow progression of their effects on holobionts complicate the elucidation of the pathogenesis of many metabolic, inflammatory, degenerative, neoplastic, and even psychiatric diseases. The interaction between specific genetic mutations, the microbiota, and environmental factors have been extensively studied in certain diseases such as Crohn’s disease, both in humans and animal models [26]. In contrast, the characteristics of the microbiota remain still unexplored in individuals with certain common genetic conditions such as Tay-Sachs, thalassemia, neurofibromatosis, and Jackson-Weiss syndrome. In other examples, such as cystic fibrosis [27], Huntington disease [28], severe combined immunodeficiency disorder [29], and sickle cell disease [30], a growing body of information is emerging on associated disturbances of the microbiota. The currently consolidating idea that interactive co-evolution of the host with its microbiota is an important driving force for evolution may predict future elucidations in the mechanisms of a holobiont becoming sick [31]. In recent years, the major roles played by environmental factors in the development of these diseases of holobionts have begun to be deciphered. Among others, these factors include overuse of antibiotics—especially at critical periods of life—cesarean sections, avoidance of breast feeding, unhealthy diets, and exposure to toxic substances. The proposed paradigm has the potential to develop novel preventive and therapeutic approaches. We suggest applying a strong ecologic attitude toward the preservation of health in all holobionts on Earth.
